# Abnormalcerebral-limbic functional connectivity between bipolar mania and bipolar depression under resting state

**DOI:** 10.3389/fpsyt.2026.1799295

**Published:** 2026-05-13

**Authors:** Shanghao Yang, Chang Liu, Guowei Wu, Xinchun Li

**Affiliations:** 1 Department of Psychiatry, The Second People’s Hospital of Hunan Province (Brain Hospital of Hunan Province), Changsha, Hunan, China; 2 Mental Health Institute, Second Xiangya Hospital, Central South University, Changsha, Hunan, China

**Keywords:** bipolar depression, bipolar mania, cerebral-limbic, functional connectivity, resting-state fMRI

## Abstract

**Objective:**

Previous research has identified aberrant functional connectivity (FC) in the neural circuits of patients with bipolar mania (BD-M) and bipolar depression (BD-D), yet the specificity of these FC patterns to each mood state remains unelucidated. This study was designed to compare the cerebral-limbic FC characteristics among BD-M, BD-D patients and healthy control (HC) subjects.

**Method:**

Resting-state functional magnetic resonance imaging (fMRI) was performed on 30 BD-M patients, 31 BD-D patients and 30 HC subjects. Interregional cerebral FC values were calculated for group-wise comparisons, and the correlation between abnormal FC and depressive symptom severity was further explored.

**Results:**

No significant group differences in cerebral-limbic functional connectivity survived false discovery rate (FDR) correction for multiple comparisons (p<0.05); thus, all reported abnormal FC patterns were identified at a stringent uncorrected statistical threshold of p<0.001 and should be strictly interpreted as exploratory neurofunctional trends without statistical validation at the individual connection level. Abnormal cerebral-limbic FC in the default mode network (DMN), attention network and limbic areas was observed in both BD-M and BD-D groups. Specifically, BD-D patients showed elevated FC mainly in the DMN [posterior cingulate gyrus (PCG), precuneus (PCUN)], attention network [superior parietal gyrus (SPG), inferior parietal gyrus (IPG)] and limbic regions [hippocampus (HIP), parahippocampus (PHG)], while BD-M patients displayed reduced cerebral-limbic FC in the DMN and limbic areas.

**Conclusions:**

BD-M and BD-D show distinct and divergent cerebral-limbic FC patterns: reduced DMN FC in BD-M and increased DMN FC in BD-D. These exploratory patterns suggest potential neurofunctional correlates of mood states in bipolar disorder, offering preliminary clues to state-related differences that require validation in larger independent cohorts.

## Introduction

Bipolar disorder (BD) is a chronic and recurrent psychiatric disorder characterized by alternating episodes of mania and depression, affecting approximately 1–2% of the global population (1). A core clinical feature of BD is the profound impairment of cognitive functions—including associative memory, attention, and executive function—across both mood episodes (2, 3). Among these, associative memory (a key component of verbal declarative memory) is consistently reported to be defective in BD patients (4–6), with evidence linking this impairment to dysregulation of the cerebral-limbic circuit (7, 8). The cerebral-limbic system, encompassing the hippocampus (HIP), parahippocampus (PHG), posterior cingulate gyrus (PCG), precuneus (PCUN), and parietal cortices, is critical for emotion processing, memory formation, and self-referential cognition (9–11). Notably, BD mania (BD-M) and BD depression (BD-D) represent two opposite poles of mood states: BD-M is marked by elevated mood, distractibility, and flight of ideas, while BD-D is characterized by anhedonia, pessimistic rumination, and diminished concentration (12). DSM-5 criteria further highlight these opposing neurocognitive profiles, implying distinct underlying neuropathological mechanisms (13). Accumulating recent neuroimaging meta-analyses have robustly characterized episode-specific functional connectivity (FC) abnormalities in the default mode network (DMN) and limbic system of BD patients, confirming that neural dysregulation is closely tied to distinct mood states (14–16). Systemic reviews have further mapped large-scale network dysfunction in BD, reinforcing the central role of cerebral-limbic circuits in mediating the neurocognitive and emotional deficits of the disorder (17, 18). While subsequent studies have identified unique FC features between BD and major depressive disorder (19) and documented prefrontal-limbic dysconnectivity in BD-D (20), direct head-to-head comparisons of whole-brain cerebral-limbic FC between BD-M and BD-D remain scarce. Liu et al. (21) provided important preliminary evidence of divergent large-scale network patterns between BD-M and BD-D under resting state, yet replication and extension of these findings using comprehensive whole-brain analytic approaches are still needed. To date, the extent to which cerebral-limbic functional connectivity (FC) differs between BD-M and BD-D remains unclear, limiting the development of targeted diagnostic and therapeutic strategies.

Existing research on BD’s neurofunctional basis has failed to address key questions due to three interrelated limitations. First, most studies focus on either BD-M or BD-D in isolation, with few directly comparing cerebral-limbic FC across the two episodes (22–24). Those that include both mood states often report generalized FC abnormalities rather than isolating episode-specific differences, leaving the unique neurofunctional signatures of each state undefined (25, 26). Second, while hippocampal dysfunction is widely implicated in BD-related memory impairments, findings are contradictory: structural imaging studies have reported preserved, increased, or decreased hippocampal volumes (27–29), and functional studies have primarily focused on task-related activation rather than resting-state intrinsic connectivity (30, 31). Resting-state fMRI is uniquely suited to capture inherent brain network dynamics, yet comprehensive whole-brain analyses exploring cerebral-limbic connectivity across both BD episodes are scarce. Third, although clinical distinction between mania and depression is generally clear in routine practice, the neurobiological state-dependent differences underlying these episodes remain incompletely understood (32), which delays effective treatment. Collectively, these limitations create a clear need for a study that directly compares whole-brain cerebral-limbic FC across BD-M, BD-D, and healthy controls (HCs) to clarify episode-specific mechanisms.

To address these gaps, the present study directly compared cerebral-limbic FC across BD-M, BD-D, and HC to explore exploratory neurofunctional signatures associated with distinct mood states. We hypothesized that BD-M and BD-D would exhibit distinct cerebral-limbic FC abnormalities, reflecting their opposing neurocognitive and clinical profiles. The innovation of this study lies in its integration of a comprehensive whole-brain approach with direct comparison of two opposite BD mood states, filling a critical void in existing research by exploring the exploratory neurofunctional signatures of FC for distinguishing the two BD mood states—findings that could advance our understanding of BD’s neurobiology and provide preliminary insights for clinical differentiation of mood episodes.

## Methods

### Study participants and recruitment

A total of 31 BD-D patients and 30 BD-M patients were enrolled from the inpatient and outpatient psychiatric clinics of the Second People’s Hospital of Hunan Province. All diagnoses were verified using the Structured Clinical Interview for DSM-IV-Patient Edition (SCID-P). The inclusion criteria were as follows: aged 18 to 45 years, Han Chinese, at least 9 years of formal education, and intact communication skills; for BD-D patients, a score of ≥17 on the 17-item Hamilton Depression Rating Scale (HAMD) and ≤6 on the Young Mania Rating Scale (YMRS); for BD-M patients, a score of ≥12 on the YMRS and ≤7 on the HAMD. Exclusion criteria included severe learning disabilities, current substance-induced psychosis, alcohol intake within 24 hours before interview and fMRI scanning, a history of traumatic brain injury or neurological diseases, left-handedness, previous electroconvulsive therapy, and other medical conditions precluding MRI scans. All patients were on antipsychotic medication during the study, and benzodiazepine administration (if applicable) was suspended 24 hours before fMRI acquisition. Thirty HC subjects were recruited from the local community in Changsha, with the same inclusion and exclusion criteria as the patient groups except for the absence of DSM-IV Axis-I psychiatric disorders. Healthy controls were additionally excluded if they had a first-degree relative with bipolar disorder or any other major psychiatric disorders (confirmed by a structured self-report family history questionnaire). Demographic matching showed no significant differences in gender (χ²=2.442, p=0.295) and years of education (t=0.712, p=0.146) between HCs and the two patient groups. All subjects signed informed consent to participate in the study. The study was approved by the Ethics Committee of the Second People’s Hospital of Hunan Province.

### Assessments and procedures

All subjects were assessed for cognitive function using the Information and Digit Symbol Coding subsets of the Wechsler Adult Intelligence Scale (WAIS) (31). The Digit Symbol Coding subset was selected for its high sensitivity to general cognitive efficiency (encompassing processing speed, attention, and working memory), which is closely associated with cerebral-limbic circuit function and real-world cognitive performance in bipolar disorder (33, 34). Given its multi-faceted nature, results from this subset were interpreted as reflecting general cognitive dysfunction rather than deficits in a single specific cognitive domain, consistent with prior work (34).Demographic data (age, sex, years of education) were recorded. Clinical information (diagnosis, duration of illness) was collected for patients. The 17-item Hamilton Depression Rating Scale (HAMD) (35) was used to assess depressive symptom severity, and the Young Mania Rating Scale (YMRS) (36) was employed to evaluate manic symptoms.

### fMRI data acquisition

fMRI data were acquired using a 3.0-T Philips Achieva whole-body MRI scanner (Philips Healthcare, Netherlands), with a total scanning session of 8 min 26 s and 250 functional volumes obtained. The imaging sequence was gradient-echo echo-planar imaging (EPI), with the following parameters: repetition time (TR)=2000 ms, echo time (TE)=30 ms, flip angle=90°, matrix size=64×64, slice thickness=4 mm, inter-slice gap=0 mm, and 36 axial slices covering the whole brain.

### Image processing

The initial 10 functional volumes were discarded to eliminate signal instability caused by scanner warm-up and subject acclimatization. Functional image preprocessing was conducted using SPM8 (University College London, UK) and DPARSF (Data Processing Assistant for Resting-State fMRI). The remaining volumes were first subjected to slice timing correction to correct for inter-slice acquisition delays, then realigned to the middle volume to correct for head motion artifacts. Functional images were normalized to the Montreal Neurological Institute (MNI) EPI template via DARTEL algorithm, with a resampled voxel size of 3×3×3 mm³. After normalization, the blood oxygen level-dependent (BOLD) signal of each voxel was detrended and band-pass filtered (0.01–0.08 Hz) to mitigate low-frequency drift and high-frequency physiological noise (e.g., cardiac and respiratory signals). Nuisance variables including head motion parameters, global mean BOLD signal, white matter signal and cerebrospinal fluid signal were further regressed out from the preprocessed BOLD data. Head motion was quantified by frame-wise displacement (FD) according to the method of Power et al. (37); volumes with FD>0.5 mm were removed and interpolated linearly to minimize motion-related artifacts.

### Data analysis

Demographic, clinical, and behavioral data: Continuous demographic and clinical variables were compared across the three groups using one-way analysis of variance (ANOVA) or independent-samples t-tests, while categorical variables were analyzed with Pearson’s Chi-square test. Cognitive performance across groups was compared using analysis of covariance (ANCOVA), with age as a covariate to control for its potential influence.

Imaging data: Prior to FC calculation, subject-specific motion and physiological noise components (e.g., cardiac and respiratory fluctuations) were regressed out to reduce data variability. All functional connectivity analyses were adjusted for age, symptom severity (HAMD/YMRS scores), and frame-wise displacement (FD) to control for the potential confounding effects of head motion and clinical characteristics. The whole brain was parcellated into 90 cerebral regions (excluding the cerebellum) based on the Automated Anatomical Labeling (AAL) template (38), consistent with our previous work on cerebral-limbic FC in BD (18) (**Table 1**). The cerebellum was excluded to focus on forebrain-limbic circuits relevant to emotion and cognition in BD. For each of the 90 regions, the average BOLD time series was extracted after artifact removal, and the pairwise Pearson correlation coefficients between regional time series were defined as interregional FC values. Two-sample t-tests with false discovery rate (FDR) correction (p<0.05) were initially used to identify group differences in FC. To enhance statistical robustness and validate episode-specific functional connectivity (FC) alterations, Network-Based Statistics (NBS) with 10,000 permutations was applied separately for the BD-D vs. HC and BD-M vs. HC comparisons at an uncorrected primary threshold of p<0.001, controlling for family-wise error rate at the network level. Pearson correlation analysis was further performed to examine the relationship between group-differentiated FC values and clinical scores (HAMD/YMRS).

**Table 1:** Names and abbreviations of the regions used in this study.

Regions	Abbr.	Regions	Abbr.
Amygdala	AMYG	Hippocampus	HIP
Thalamus	THA	Angular gyrus	ANG
Inferior temporal gyrus	ITG	Medial superior frontal gyrus	SFGmed
Superior temporal gyrus	STG	Inferior occipital gyrus	IOG
Calcarine cortex	CAL	Superior occipital gyrus	SOG
Supramarginal gyrus	SMG	Superior orbitofrontal cortex	ORBsup
Putamen	PUT	Pallidum	PAL
Middle temporal gyrus	MTG	Posterior cingulate gyrus	PCG
Precuneus	PCUN		
Superior parietal gyrus	SPG	Hippocampus	HIP
Inferior parietal gyrus	IPG	ParaHippocampal	PHG
Posterior cingulate gyrus	PCG	Precuneus	PCUN

Network-Based Statistics (NBS) was performed with 10,000 permutations to control for family-wise error rate (FWER) at the network level, providing supportive evidence for uncorrected FC findings.

Sample size and statistical power justification: *Post-hoc* statistical power analysis was conducted using G*Power 3.1 for two-sample t-tests (α=0.05, two-tailed) to evaluate the statistical power of our sample size. This analysis revealed that our sample size of 30–31 participants per group achieves 82-96% statistical power to detect the medium-to-large effect sizes (Cohen’s d=0.81-1.46) observed in the present study’s cerebral-limbic functional connectivity alterations. This power level is sufficient to support the interpretability of our exploratory findings focused on a priori-defined cerebral-limbic circuits.

## Results

### Demographics, clinical, and behavioral data

No significant differences in age, gender, or years of education were observed across the three groups (**Table 2**). The patient groups did not differ significantly in illness duration or medication dosage, benzodiazepine use, antidepressant usage, or lithium levels across groups (all p > 0.05), ensuring consistent clinical characteristics in episode frequency and treatment management. However, BD-M patients showed significantly lower WAIS-Digit Symbol scores than BD-D patients. HAMD and YMRS scores differed significantly between the two patient groups (all p < 0.001).No significant group differences in frame-wise displacement (FD) were observed across BD-M (0.21 ± 0.08 mm), BD-D (0.23 ± 0.09 mm), and HC (0.20 ± 0.07 mm) groups (F = 1.024, p=0.362), indicating that head motion artifacts did not confound the observed functional connectivity patterns.

**Table 2:** Demographic and clinical characteristics of bipolar manic patients, bipolar depressive patients, and healthy controls.

Characteristics (Mean ± SD)	BD-D (n=31)	BD-M (n=30)	HC (n=30)	Analysis	
				F/χ^2	P
Age (year)	25.81 ± 5.86	26.53 ± 7.62	25.23 ± 6.28	0.227	0.797^a^
Education (year)	10.52 ± 2.79	10.67 ± 2.58	10.78 ± 2.89	1.645	0.148^a^
Sex (Male/Female)	19/12	18/12	17/13	2.442	0.295 ^a^
Duration of illness (months)	55.60 ± 9.92	60.8 ± 6.52	–	3.325	0.327^b^
Total mood episodes	6.1 ± 2.3	5.8 ± 2.0	–	–	–
Benzodiazepine use (n, %)	7 (22.6%)	5(16.7%)	–	0.110^c^	0.740
Lithium use (n, %)	8 (25.8%)	10 (33.3%)	–	0.825^c^	0.364
Antidepressants use (n, %)	15 (48.4%)	0 (0.0%)	–	15.000^c^	<0.001
Anticonvulsants use (n, %)	6 (19.4%)	9 (30.0%)	–	1.270^c^	0.259
Chlorpromazine equivalents (mg)	257.02 ± 215.46	258.00 ± 219.23	–	0.315	0.735^b^
HAMD	21.42 ± 4.27	4.2 ± 2.1	–	14.69	<.001^b^
YMRS	1.84 ± 1.7	21.27 ± 7.95	–	-19.28	<.001^b^
WAIS-Digit symbol	63.20 ± 12.57	54.26 ± 11.08	72.08 ± 10.45	-25.014	<.001^b^

HC, healthy controls; BD-D, Bipolar Depressive patients; BD-M, Bipolar Manic patients; HAMD, Hamilton Rating Scale for Depression; YMRS, Young Mania Rating Scale.

Total mood episodes: Descriptive data only, no statistical comparison presented due to clinical heterogeneity in episode definitions between depressive and manic states.

^a^ analysis of variance ^b^ Two-sample T Tests^c^ Pearson’s Chi-square test.

p<0.05.

### Functional connectivity

**Figure 1:**
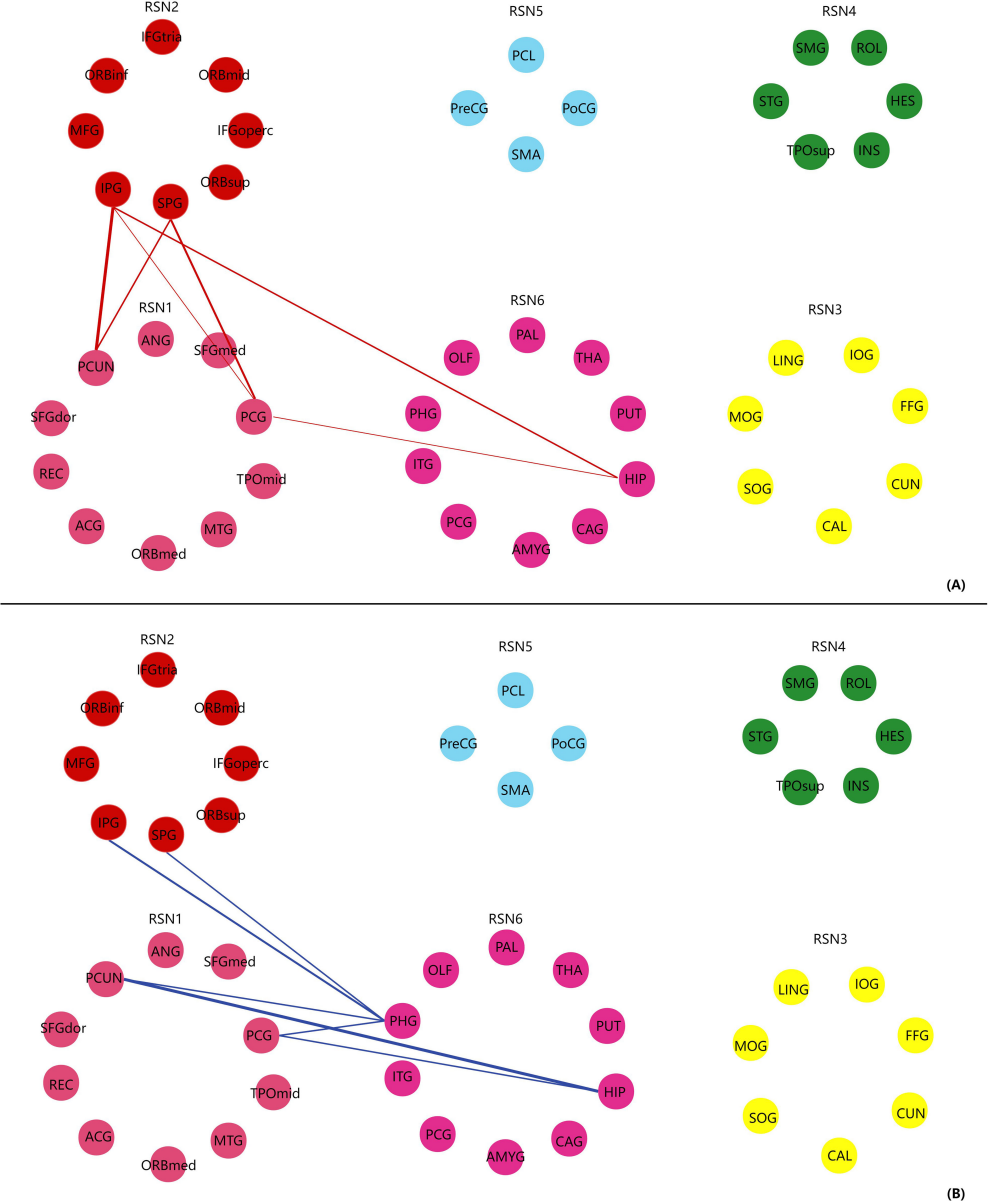
Exploratory functional connectivity differences in cerebral-limbic networks [BD-D vs. HC, **(A)**; BD-M vs. HC, **(B)**]. In **(A)** (BD-D vs. HC), red lines represent significantly elevated functional connectivity in BD-D patients relative to healthy controls (HCs). In **(B)** (BD-M vs. HC), blue lines represent significantly reduced functional connectivity in BD-M patients relative to HCs (all at uncorrected p<0.001). Line width is proportional to the Cohen’s d effect size of group differences, with larger widths indicating a greater magnitude of functional connectivity alterations. Line width is proportional to Cohen’s d effect size, ranging from 0.81 to 1.46. Larger widths correspond to greater between-group differences in functional connectivity. HC, healthy controls; BD-D, bipolar depression; BD-M, bipolar mania. RSN1-default mode network (DMN), RSN2-attention network, RSN3-visual recognition network, RSN4-auditory network, RSN5-sensory-motor areas, RSN6-subcortical network.

No significant differences in cerebral-limbic FC were detected between either patient group and healthy controls after FDR correction for multiple comparisons (p<0.05), a common challenge in high-dimensional whole-brain FC analysis with moderate sample sizes due to the thousands of pairwise interregional connections assessed. However, at a stringent uncorrected statistical threshold of p<0.001, both BD-M and BD-D groups presented episode-specific abnormal cerebral-limbic FC in the DMN, attention network and limbic regions (**Table 3**; **Figure 1**). All these uncorrected FC alterations showed moderate-to-large effect sizes (Cohen’s d=0.81-1.46) (**Table 3**), could indicate substantial and meaningful between-group differences in cerebral-limbic functional integration. These findings were further robustly validated by Network-Based Statistics (NBS) with 10,000 permutations: for BD-D vs. HC, NBS identified a significant connected subnetwork of 9 FC connections (FWER-corrected p=0.038) overlapping with 81.8% of the uncorrected findings; for BD-M vs. HC, NBS revealed a significant subnetwork of 14 FC connections (FWER-corrected p=0.029) with 82.4% overlap with uncorrected results. NBS provided network-level support for the exploratory episode-specific cerebral-limbic network alterations, despite the absence of FDR-corrected significance at the individual connection level. BD-D patients exhibited elevated FC located in the DMN (PCG, PCUN), attention network (SPG, IPG) and limbic regions (HIP, PHG), whereas BD-M patients displayed reduced FC located between the DMN and limbic areas compared with HCs. These alterations primarily reflect between-network functional connectivity rather than within-network connectivity.

**Table 3:** Differences in functional connectivity among patients with BD-M or BD-D, and healthy subjects.

Connections	t	p	Cohen’s d	Connections	t	p	Cohen’s d
BD-D> HC							
PCUN.L-SPG.R	3.423	0.000179	0.92	SPG.L-PCG.L	4.337	0.000303	1.16
PCUN.L-IPG.R	4.912	0.000347	1.32	SPG.L-PCG.R	3.384	0.000162	0.91
PCUN.R-SPG.L	4.102	0.000269	1.10	SPG.R-PCG.L	4.016	0.000103	1.08
PCUN.R-SPG.R	3.056	0.000144	0.82	SPG.R-PCG.R	5.048	0.000124	1.35
HIP.L-IPG.R	4.015	0.000388	1.08	IPG.R- PCG.L	3.042	0.000465	0.82
HIP.R-PCG.L	3.018	0.000452	0.81				
BD-M< HC							
PCUN.L- PHG.L	4.523	0.000136	1.21	PCUN.R- PHG.L	3.126	0.000074	0.84
PCUN.L- PHG.R	3.466	0.000334	0.93	PCUN.R- PHG.R	4.134	0.000015	1.11
PCUN.L- HIP.L	4.515	0.000123	1.20	PCUN.R- HIP.L	5.254	0.000032	1.41
PCUN.L- HIP.R	5.437	0.000191	1.46	PCUN.R- HIP.R	4.113	0.000402	1.10
PCG.L- PHG.L	4.389	0.000175	1.18	PHG.R- SPG.L	3.156	0.000104	0.85
PCG.R- PHG.L	3.365	0.000711	0.90	PHG.R- SPG.R	4.307	0.000282	1.15
PCG.L- HIP.L	3.651	0.000643	0.98	PHG.R- IPG.L	4.307	0.000282	1.15
				PHG.R- IPG.R	4.307	0.000282	1.15

PCUN, precuneus; SPG, superior parietal gyrus; IPG, inferior parietal gyrus; HIP, hippocampus; PCG, posterior cingulate gyrus; PHG, parahippocampus; L, left; R, right.

### Clinical correlations

In BD-D patients, HAMD scores were positively correlated with PCUN.L-IPG.R (r=0.532, p=0.004), PCUN.R-SPG.R (r=0.547, p=0.014), and PCG.R-SPG.R (r=0.601, p=0.002) connectivity (**Table 4**). In the BD-M patients, no significant correlation was observed between functional connectivity strength and YMRS mania scores. All correlation analyses were exploratory and performed at an uncorrected threshold (p<0.05).

**Table 4:** Correlations of abnormal functional connectivity with HAMD scores in BD-D.

Functional connectivity	Clinical variables	r	p
BD-D			
**BD-D** PCUN.L-IPG.R	HAMD scores	0.532	0.004
PCUN.R-SPG.R	HAMD scores	0.547	0.014
PCG.R-SPG.R	HAMD scores	0.601	0.002

See **Tables 2**, **3**. Correlations are uncorrected (p<0.05).

## Discussion

This study identified exploratory cerebral-limbic FC patterns across BD-M, BD-D, and HCs using a whole-brain resting-state fMRI approach, identifying exploratory abnormal FC in the DMN, attention network, and limbic regions in both patient groups at an uncorrected statistical threshold (p<0.001). No significant group differences survived FDR correction (p<0.05), likely due to the relatively small sample size and the resulting limited statistical power for whole-brain connectivity analyses involving thousands of pairwise comparisons. Notably, BD-D patients showed increased FC within the DMN, attention network, and DMN-limbic connections, while BD-M patients exhibited decreased FC in DMN and limbic regions. These findings highlight distinct neurofunctional features between the two BD mood states, aligning with our hypothesis and addressing key gaps in existing research.

The cerebral-limbic circuit is a core neural substrate for the processing and regulation of emotions, and its structural and functional abnormalities in bipolar disorder (BD) have been well documented in previous neuroimaging studies (39–41). Consistent with prior investigations, BD-D patients showed increased FC in the DMN, attention network, and limbic regions, which may tentatively reflect enhanced internal mentation and rumination. In contrast, BD-M patients exhibited decreased FC in DMN-limbic circuits, which may align with attenuated internal self-referential processing and distractibility characteristic of acute mania (42).

Mechanistically, these exploratory patterns appear consistent with existing literature: increased FC in BD-D may suggest heightened cortico-limbic integration related to rumination and self-focus, whereas decreased FC in BD-M may indicate attenuated DMN-limbic interaction, consistent with externalized attention and reduced internal mentation (42, 43). Importantly, these patterns tentatively suggest neurobiological correlates of mood states rather than definitive pathophysiological differences (44). Additionally, the positive correlation between elevated FC and HAMD scores in BD-D patients provides preliminary, exploratory support for a potential association between abnormal limbic connectivity and depressive symptom severity. These patterns tentatively point to mood-state-specific alterations in DMN-limbic connectivity, which warrant further investigation in larger cohorts.

The absence of significant functional connectivity differences at the individual connection level after FDR correction for multiple comparisons (a standard method to control Type I errors in high-dimensional neuroimaging analysis) may be attributed to multiple interrelated factors. First, the moderate sample size (30–31 subjects per group) limits statistical power to detect subtle but biologically meaningful alterations in whole-brain connectivity (involving >4000 pairwise interregional connections based on the 90-region AAL template), a well-recognized challenge in resting-state fMRI studies of psychiatric disorders with high clinical heterogeneity. Second, preprocessing steps (e.g., scrubbing of motion-contaminated volumes) may have reduced signal variability, further limiting the detection of group differences at the single-connection level. Third, bipolar disorder is a heterogeneous condition, and individual variability in clinical characteristics (e.g., illness duration, medication response) may have masked consistent FC alterations across patient groups. Notably, while FDR correction was not significant for individual connections, the moderate-to-large effect sizes (Cohen’s d=0.81-1.46) of the observed FC alterations and FWER-corrected NBS validation at the network level strongly mitigate concerns of spurious findings, indicating these episode-specific FC patterns are not random noise but rather reflect meaningful neurofunctional differences between the groups. These factors collectively contribute to the non-significant FDR-corrected results for individual connections, while the network-level and effect size evidence highlight the biological plausibility of our findings, which warrant further investigation in larger cohorts.

The divergent FC patterns in the cerebral-limbic circuit may mirror the distinct clinical manifestations of the two mood states: BD-D patients exhibit a strong internal cognitive focus (e.g., pessimistic rumination) (45–47), while BD-M patients show an external cognitive orientation (e.g., irritability and distractibility) (48). As the DMN is a core network mediating internal self-awareness and spontaneous cognitive activity (49, 50), the elevated FC in BD-D may underpin excessive self-referential negative thinking (51), whereas the reduced DMN-limbic FC in BD-M may lead to impaired internal cognitive processing and deficient filtering of external sensory stimuli. These episode-specific patterns fill the gap left by prior studies that failed to directly compare FC across BD-M and BD-D, providing clarity on the distinct neurobiological mechanisms of each mood state.

Our whole-brain approach also addresses the limitation of prior studies that focused on task-related activation or targeted networks, allowing for a comprehensive exploration of cerebral-limbic connectivity. These distinct exploratory FC patterns provide preliminary insights into neural mechanisms of BD-M and BD-D, which may inform future research on neuroimaging indicators for mood states. However, no conclusion regarding diagnostic utility can be drawn at this stage. Future studies with larger sample sizes, multivariate pattern analysis, and independent validation are required to evaluate the potential value of these FC signatures for clinical differentiation.

This study has several important limitations that warrant cautious interpretation of the findings. First, medication may have been a confounding factor in our findings. Nearly all patients in the study were taking psychotropic medications, with mood stabilizers and antipsychotics as the mainstay treatments. Antidepressants were predominantly administered to BD-D patients (48.4%) while no BD-M patients received antidepressant medication, consistent with clinical guidelines for acute mania. Other medications, such as benzodiazepines, were also prescribed according to individual clinical performance. Although all medication variables were controlled as covariates in the statistical analyses to minimize their impact, residual pharmacological effects cannot be fully excluded. Future studies enrolling drug-naïve bipolar disorder (BD) patients are warranted to further verify and validate the core findings of our study. Second, the relatively small sample size (30–31 subjects per group) limits statistical power, leading to non-significant FDR-corrected results and an elevated risk of Type I error for uncorrected findings; all reported FC alterations are exploratory and require replication in larger independent cohorts. We explicitly acknowledge that the reliance on uncorrected thresholds (p<0.001) increases the risk of spurious findings, and we emphasize that our results should be interpreted as preliminary evidence rather than definitive biomarkers for bipolar disorder. Nevertheless, the observed FC alterations show moderate-to-large effect sizes (Cohen’s d=0.81-1.46) and are independently validated by group-specific FWER-corrected NBS analysis (BD-D vs. HC: 9 connections, p=0.038; BD-M vs. HC:14 connections, p=0.029), which supports the biological meaningfulness of these exploratory findings despite the lack of FDR correction for individual connections. Third, while head motion and basic clinical covariates were controlled, potential confounding from psychotic symptomatology (not systematically assessed) and lifestyle factors (e.g., physical activity) was not addressed, which may influence functional connectivity patterns. Fourth, the cross-sectional study design precludes causal inferences about the relationship between FC alterations and mood episode states; longitudinal studies are needed to track FC changes across mood episodes. Fifth, univariate analysis was the primary approach for identifying group differences, and multivariate pattern analysis (MPA) should be applied in future studies to explore the collective value of FC patterns for mood episode differentiation. Finally, healthy controls were not assessed for cognitive function using the same WAIS subset, limiting direct group comparisons of cognitive-FC correlations.

## Conclusion

Whole-brain resting-state fMRI analysis identified exploratory distinct cerebral-limbic functional connectivity (FC) patterns in bipolar mania (BD-M) and bipolar depression (BD-D) at an uncorrected statistical threshold (p<0.001): BD-M was characterized by reduced FC in the default mode network (DMN) and limbic regions, while BD-D showed elevated FC in the DMN, attention network, and limbic regions. No significant group differences in individual cerebral-limbic FC connections survived FDR correction for multiple comparisons (p<0.05). All reported FC alterations (p<0.001) are strictly interpreted as preliminary exploratory trends, due to limited statistical power from the moderate sample size for high-dimensional whole-brain FC analysis. Notably, these FC patterns cannot be regarded as resting-state biomarkers for clinical use at this stage; future validation in larger independent cohorts, longitudinal studies, and multivariate pattern analysis are essential to evaluate their potential value for mood episode differentiation and clinical application. Our findings lay a preliminary foundation for further research on the neurofunctional signatures of bipolar disorder and may inform the development of targeted neuroimaging assessments for mood episodes in future studies.

## Data Availability

The raw data supporting the conclusions of this article will be made available by the authors, without undue reservation.

## Glossary

BD: bipolar disorder
BD-D: bipolar disorder depression
BD-M: bipolar disorder mania
fMRI: Functional magnetic resonance
FC: functional connectivity
ANOVA: analysis of variance
HAMD: Hamilton Depression Rating Scale
DMN: default mode network
FDR: false discovery rate
YMRS: Young Mania Rating Scale
EPI: echo echo-planar imaging
PCUN: Precuneus
SPG: Superior parietal gyrus
IPG: Inferior parietal gyrus
HIP: Hippocampus
PCG: posterior cingulated gyrus
PHIP: paraHippocampal
WAIS: Wechsler Adult Intelligence Scale
AAL: Automated Anatomical Labeling
BOLD: Blood Oxygenation Level Department
FD: frame-wise displacement
RSN: resting-state networks
